# Is blood transfusion safe during the COVID-19 pandemic?

**DOI:** 10.2144/fsoa-2020-0116

**Published:** 2020-09-17

**Authors:** Josiane Bassil, Elie Rassy, Joseph Kattan

**Affiliations:** 1 Hematology-Oncology Department, Hotel Dieu de France University Hospital, Faculty of Medicine, Saint Joseph University, Beirut, Lebanon

**Keywords:** blood products, coronavirus, COVID-19, transfusion, transmission

The rapid spread of the coronavirus disease 2019 (COVID-19) pandemic has challenged healthcare systems to re-organize the management of patients in a short period [1]. The causative pathogen of this pandemic is severe acute respiratory syndrome coronavirus 2 (SARS-CoV-2), one of seven coronaviruses that can be found in humans. It is a rapidly mutating, enveloped, ssRNA, beta-coronavirus [2,3]. The initial reports published after the pandemic spread showed a transmissibility rate of 2.2 and possibly low values of dispersion. Subsequently, its pattern of human-to-human transmission is rather similar to the international spread of SARS-CoV, which presents a sustained human-to-human transmission and occassional super spreading events [4]. COVID-19 spreads mainly through droplets during close contact with a symptomatic patient (sneezing, coughing for example) and may be airborne during direct exposure to the virus such as endotracheal intubation, bronchoscopy, open suction, nebulized treatment, manual ventilation before intubation, mechanical ventilation, tracheostomy and cardiopulmonary resuscitation. The virus has a cytopathogenic effect on epithelial cells in the human airway and alveolar cells. Upon exposure, the virus becomes attached to angiotensin-converting enzyme 2 and transmembrane protease serine 2 that are expressed mainly in bronchial transient secretory cells via its spike proteins, entering the cell whereupon there is RNA translation and a series of events after which the virus is released as virion inside the cell [5]. As the available evidence remains weak, the medical community is attempting to close gaps in our knowledge of COVID-19 – including the transmissibility of the virus during blood transfusions.

Blood transfusion is an almost routine medical procedure that is commonly life-saving, repleting blood losses due to surgery, injury and chemotherapy. In practice, 15% of the daily transfused units of packed red blood cells are allocated to hematology and oncology patients [6]. Moreover, almost a third of patients with major cardiac surgeries will require postoperative transfusions [7]. The COVID-19 pandemic has impacted medical care among these patients as blood donations were limited during the lockdown period. Mobility constraints have reduced the number of blood donations and supplies and the safety of blood transfusion was a matter of considerable concern [8]. The backdrop for these fears was the presence of plasmatic SARS-CoV-2 RNA in 15% of symptomatic patients with COVID-19 as reported by the American Association of Blood Banks and the possibility of blood transmission as reported in vertical mother-to-fetus transmission in pregnant COVID-19-infected women [9,10]. However, the transmission of SARS-CoV-2 is not systematic; other case series reported on negative tests in fetuses of COVID-19-infected mothers [11,12]. In this Editorial we provide an overview of the blood transfusion policies taken to ensure the safety of the recipients from COVID-19.

## Current clinical practice in the absence of guidelines

In the absence of evidence-based guidelines, the international community has adopted conservative measures to ensure the safety of blood products at the level of blood donation [13,14]. The recommendations suggest the education of donors on self-deferring in exposed or symptomatic patients and informing the blood center if they develop COVID-19-related symptoms within 28 days of donation. Patients who had or were exposed to COVID-19 had to self-defer for at least 28 days. It was also recommended to quarantine the blood products until the donor is considered safe defined by the ‘absence of a reported subsequent illness’ and recall the blood components if the donor became symptomatic or reported contact with a certified case within 14–28 days of donation [13,14].

These precautions are very effective in avoiding blood products that are potentially contaminated from symptomatic donors but cannot overcome the possibility of having blood donations from asymptomatic patients. The available screening tests are not yet reliable and remain an unmet need before blood donation. Viral RNA, tested by PCR and a nasopharyngeal swab, is likely to be undetectable before the onset of symptoms. Similarly, the serologic test is also negative in asymptomatic donors with seroconversion occurring between the 3rd and 4th week of symptom onset [15]. Moreover, screening the blood product coming from asymptomatic persons has not been recommended [13,14]. The published literature has reported on the occurrence of blood transfusions from COVID-19-infected patients in several instances. The transfusion of platelets from a donor revealed to be infected with COVID-19, 3 days after platelet donation to a patient diagnosed with severe aplastic anemia did not contaminate the recipient, who did not develop any symptoms of COVID-19 [16]. Similarly, blood and platelet transfusion to nine recipients from COVID-19-positive donors who donated before they became symptomatic (a minimum of 3 days interval between the blood donation and the onset of symptoms), did not transmit the virus to the recipients [17]. The viral load of asymptomatic patients may be insufficient to transmit the infection to the blood recipient or may be neutralized during certain blood manipulations. In this regard, the preparation of convalescent plasma units from patients with confirmed COVID-19 infections used methylene blue and visible light in an attempt to protect against the transmission of the virus [18]. The addition of riboflavin to plasma and whole blood followed by exposure to ultraviolet light was shown to be a useful method in reducing the infectivity of SARS-CoV-2 [19]. It is effective in reducing infectious viral loads and making the spike protein adsorption and cellular entry very difficult. Nevertheless, the guidelines have not recommended COVID-19 eradication [13,14].

The prescription of blood products has been impacted by the decision of the medical community to defer elective surgeries and confinement, which decreased the number of traumatic accidents. The strict adherence to restrictive transfusions limited liberal transfusions but still, several centers have had blood product shortages, unable to even provide urgent blood transfusions [20].

Cancer centers have tried to avoid inpatient stays, giving less myelosuppressive and intensive chemotherapy in hematologic disease, with increased prescription of growth factors and shifting, whenever possible, to an outpatient infusion center, with less frequent follow-up and laboratory monitoring [21–23]. They have turned to supportive drugs, such as erythropoietin and thrombopoietin mimetics, to limit blood support. When indicated, the allogeneic stem cell transplant is to be pursued in relapsed acute myeloid leukemia and after second complete remission patient with acute lymphoblastic leukemia, only if the patient is negative for COVID-19. In patients with solid cancers, neoadjuvant chemotherapies have been favored with the postponement of elective surgeries part of several policies, in order to limit the number of visitors, and screening of cancer patients for COVID-19 [21].

## Future recommendations

Although the transmission of COVID-19 during blood transfusion has not been reported yet, its occurrence remains possible given vertical blood transmission from pregnant women to their newborns has been reported. Avoiding the contaminated donor seems a successful strategy that has limited blood transmission; however, this may fail to avoid asymptomatic patients. Subsequently, blood banks are endeavoring to avoid blood products coming from revealed positive donors as well as exposed and asymptomatic donors. However, as screening tests are unreliable at the donor or blood donation level, the blood preparation process may be the best safety checkpoint to ensure that the transfusion is not contaminated.

Many questions remain unanswered, particularly how long individuals with previous SARS-CoV-2 infections should be considered ineligible for blood donations, in light of the progressive return to the normal daily practice and increasing demand on blood products. For this reason, the increasing number of asymptomatic infected patients should be used as an opportunity to fill the knowledge gap reharding the transmission of the virus in the blood. We propose the start of prospective trials by testing the recipients of blood products for SARS-CoV-2, or assessing donor blood to determine infectivity. One recent trial examined sputum, oral swabs and blood samples from a small cohort of 18 symptomatic and asymptomatic donors with SARS-CoV-2 infection using RT-PCR and RNA in blood testing in order to assess the risk of transfusion-related transmission, showing no risk of transmission [24]. Completing this study with a larger population and long-term follow-up on asymptomatic patients and the blood receivers will create a dynamic database. This strategy will help direct further investigations into understanding the pathogenesis of the new virus, in the absence of the blood transmission of previous, related coronaviruses [25–27].
